# Importance of early postoperative mobilization: comprehensive review

**DOI:** 10.1093/bjsopen/zrag016

**Published:** 2026-03-24

**Authors:** Abdulaziz Alsuwaylihi, Dominic O’Connor, Girish P Joshi, Henrik Kehlet, Dileep N Lobo

**Affiliations:** Nottingham Digestive Diseases Centre, Division of Translational Medical Sciences, School of Medicine, University of Nottingham, Queen’s Medical Centre, Nottingham, UK; National Institute for Health Research Nottingham Biomedical Research Centre, Nottingham University Hospitals and University of Nottingham, Queen’s Medical Centre, Nottingham, UK; Department of Clinical Nutrition, King Saud Medical City, Ministry of Health, Riyadh, Saudi Arabia; School of Health Sciences, University of Nottingham, Queen’s Medical Centre, Nottingham, UK; Department of Anesthesiology and Pain Management, University of Texas Southwestern Medical Center, Dallas, Texas, USA; Section for Surgical Pathophysiology, Rigshospitalet, Copenhagen University, Copenhagen, Denmark; Nottingham Digestive Diseases Centre, Division of Translational Medical Sciences, School of Medicine, University of Nottingham, Queen’s Medical Centre, Nottingham, UK; National Institute for Health Research Nottingham Biomedical Research Centre, Nottingham University Hospitals and University of Nottingham, Queen’s Medical Centre, Nottingham, UK; MRC Versus Arthritis Centre for Musculoskeletal Ageing Research, School of Life Sciences, University of Nottingham, Queen’s Medical Centre, Nottingham, UK; Divison of Surgery, Perelman School of Medicine, University of Pennsylvania, Philadelphia, Pennsylvania, USA

**Keywords:** perioperative care, ambulation, enhanced recovery after surgery, pathophysiology, barriers, enablers

## Abstract

**Background:**

Early postoperative mobilization is an important component of enhanced recovery after surgery protocols, which aim to improve postoperative outcomes. This narrative review explores the historical evolution, physiological impact, and clinical advantages of early postoperative mobilization.

**Methods:**

The Embase, MEDLINE, and PubMed databases were searched, without time restrictions, for studies related to postoperative immobilization and mobilization. Randomized clinical trials, observational studies, systematic reviews, meta-analyses, and clinical guidelines pertaining to adult surgical patients were reviewed, aiming to summarize the historical background, the pathophysiology of immobilization, the clinical outcomes of early postoperative mobilization, anaesthetic aspects, adverse events, limitations of the current evidence, and key barriers.

**Results:**

Extended postoperative immobilization was consistently linked with negative physiological outcomes, including muscle atrophy, insulin resistance, venous thromboembolism, and postoperative pulmonary complications. Several studies indicated that each additional day of postoperative bed rest was associated with a nearly threefold increase in the risk of postoperative pulmonary complications, and remaining on bed rest for more than 3 days was associated with a 2.7-fold higher risk of postoperative pneumonia. Conversely, early postoperative mobilization was associated with shorter hospital length of stay, with reductions of up to 34% reported in some surgical populations, and improved functional recovery. However, the effects of early postoperative mobilization on morbidity, quality of life, and mortality were inconsistent across studies.

**Conclusions:**

Early postoperative mobilization provides significant functional and physiological advantages. However, the strength of evidence supporting its impact on clinical outcomes varies according to surgical subspecialities. To improve implementation and reinforce evidence-based recommendations, future research should focus on standardized definitions of early postoperative mobilization, consistent outcome measures, and higher-quality, subspeciality-specific studies.

## Teaser

This narrative review explores the historical evolution, physiological impact, and clinical advantages of early postoperative mobilization. Early mobilization has been associated with reduced hospital length of stay, lower postoperative pain scores, fewer complications, and better functional recovery. However, the recent evidence regarding the impact of early mobilization on postoperative complications and overall clinical outcomes is inconsistent.

## Introduction

Early postoperative mobilization is an important component of enhanced recovery after surgery (ERAS) programmes^1^, as well as that of DREAMing (drinking, eating and mobilizing)^2^, but there has been little discussion of definitions or the documentation of the effect of early postoperative mobilization on outcomes.

The deleterious effects of immobilization are well established at the molecular, tissue, and functional levels^3^, and the logical argument is that early postoperative mobilization would help improve patient physiology and clinical outcomes. However, several factors, including patient characteristics (for example, advanced age, high and low body mass index, malnutrition, frailty, disability, and cognitive dysfunction), suboptimal pain management, orthostatic intolerance, opioid-related adverse effects (for example, nausea, vomiting, dizziness, and oversedation), delirium, surgical and medical complications, and traditional principles of care, may impede the implementation of early postoperative mobilization^4^. The presence of catheters (for example, urinary and epidural), tubes, and drains (for example, nasogastric and wound drains) may also delay mobilization.

The aim of this narrative review was to examine the historical development of early in-hospital postoperative mobilization, its physiological effects, and facilitators and barriers to implementation. The aim was also to establish whether early postoperative mobilization was associated with improved clinical outcomes, such as decreased postoperative complications, shortened hospital length of stay (LOS), enhanced quality of life (QoL), increased patient satisfaction, reduced readmission rates, decreased mortality, and lower overall healthcare costs.

## Methods

### Search strategy and selection criteria

A literature search was performed on 25 February 2025 and updated on 8 October 2025 within the Embase, MEDLINE, and PubMed databases. The search included all relevant publications without specific time limits, using the terms ‘postoperative’, ‘immobilization’, ‘early mobilization’, ‘early discharge’, and ‘major surgery’, in combination with words under ‘postoperative care’, ‘preoperative care’, ‘bed rest’, ‘early ambulation’, ‘length of stay’, ‘deep vein thrombosis’, ‘DVT’, ‘atrophy’, ‘musculoskeletal’, and ‘metabolic’.

This research included studies in adult surgical patients, focusing on randomized clinical trials (RCTs), meta-analyses, systematic reviews, cohort studies, and clinical guidelines. This review is divided into sections covering the historical background of immobilization and early postoperative mobilization, the pathophysiology of immobilization, the clinical outcomes of early postoperative mobilization, perioperative and anaesthetic considerations, adverse events, limitations of the current evidence, and key barriers to and enablers of implementation.

Priority was given to studies that compared the effect of early mobilization with one or more comparators on clinical outcomes. Works published after 2015 were prioritized to reflect current practice, and the quality of the included RCTs was assessed using the Cochrane Collaboration Risk of Bias tool (RoB2)^5^. Studies on paediatric or non-surgical populations and those focusing solely on regional mobilization were excluded.

## Results and Discussion

### History of immobilization and early postoperative mobilization

The earliest documented benefit of early postoperative mobilization was by Ephraim McDowell in 1819 on a patient who underwent ovarian surgery^6^. The benefits of early mobilization (adopted by the patient without medical advice) were evidenced by her engaging in light daily activities, such as making her bed, 5 days after surgery. In addition, early mobilization accelerated the patient’s recovery, enabling her to travel 70 miles on horseback just 25 days after surgery^6^. However, the work of McDowell^6^ was criticized by Michener and Henderson^6^, who indicated that these activities should not have occurred, because the patient should have had bed rest.

In the late 1890s and early 1900s, Ries^7^ and Boldt^8^ emphasized the importance of early mobilization after abdominal surgery to prevent muscle atrophy and promote rapid recovery. They argued that strict bed rest was unnecessary, and that gradual early movement prevented muscle atrophy, aided recovery of strength, and enabled discharge around 12 days after surgery. Ries^7^ and Boldt^8^ challenged extended postoperative immobilization and demonstrated improved outcomes, by reducing the risk of wound and postoperative complications, such as venous stasis and lung congestion, leading some practitioners to adopt similar methods.

Early postoperative mobilization was recognized as a critical component of postoperative recovery in the 1940s, and it was suggested that early mobilization, initiated on the first postoperative day, may reduce complications such as pneumonia and thrombophlebitis^9–11^. When commenced within 24 hours (h) after anaesthesia, early mobilization also improved pulmonary recovery with minimal side-effects, thereby enhancing patient morale^9,10,12^. Early and progressive postoperative mobilization has been associated with accelerating recovery and minimizing the duration of bed rest for specific populations, such as patients undergoing cardiac and orthopaedic surgery^13–15^. The historical evolution of early postoperative mobilization is summarized in *Table S1*.

### Pathophysiological consequences of immobilization and impact on postoperative recovery

**Fig. 1:**
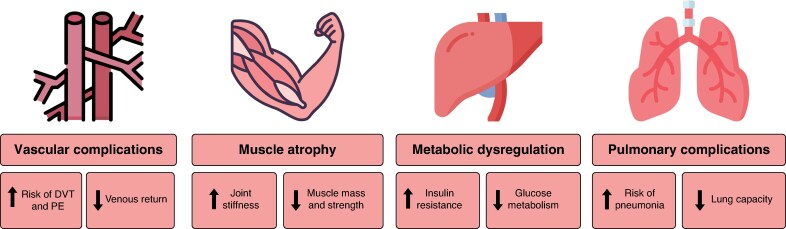
Pathophysiological consequences of immobilization DVT, deep vein thrombosis; PE, pulmonary embolism.

Prolonged postoperative immobilization has been associated with adverse effects (*Fig. 1*), such as pulmonary, musculoskeletal, metabolic, and cardiovascular complications^12,16^, leading to delayed recovery, increased morbidity, and prolonged LOS.

Immobilization of a single limb or the entire body may result from injury, disease, frailty, or surgical procedures^17–19^. Similar physiological changes may also be seen in situations involving mechanical unloading, such as during spaceflight, where microgravity rather than physical immobilization leads to reduced skeletal loading^20^, or simply due to diminished physical activity^21^. Although immobilization can occur at various stages of the lifespan, it is more prevalent in older adults, often due to chronic illness, falls, or hospitalization, associated with cumulative functional decline^22^. Regardless of the cause, immobilization reduces muscular function and muscle mass due to the mechanical unloading of the immobilized musculature. This leads to a diminished ability to perform activities of daily living and a decline in QoL^23^.

#### Vascular consequences

Deep vein thrombosis (DVT) predominantly originates in the lower limb and pelvic veins. Genetic predispositions and various acquired conditions, such as major surgery, trauma, malignancies, pregnancy, and immobilization, play significant roles in the pathogenesis of DVT^24^. Prolonged immobilization leads to venous stasis, a significant component of Virchow's triad (the other two components being endothelial damage and hypercoagulability), all of which lead to thrombus formation. A prolonged lack of muscle contraction impairs the calf muscle pump, leading to decreased venous return and a risk of thrombosis^25^.

Studies have found that the prevalence of DVT linked to orthopaedic surgery ranges from 40 to 60% without prophylactic measures, with total hip or knee arthroplasty procedures exhibiting a higher risk than less invasive orthopaedic procedures; pulmonary embolism may affect up to 20% of these patients^26,27^.

In the intensive care unit (ICU) setting, proximal DVT and isolated distal DVT were found in 14.3 and 15.5% of 252 immobile patients with mixed medical diagnoses (including leg trauma), respectively, compared with 4.4% and 10.5% of 248 mobile patients, respectively^28^. Whereas the risk of isolated distal DVT did not differ significantly between mobile and immobile patients (odds ratio (OR) 1.56, 95% c.i. 0.92–2.66; *P* = 0.111), the likelihood of developing proximal DVT was considerably higher in immobile patients (OR 3.59, 95% c.i. 1.76–7.23; *P* = 0.0001)^28^.

A systematic review comprising 15 studies and 80 678 patients with lower limb trauma confirmed that prolonged immobilization increased both the risk of both symptomatic and asymptomatic venous thromboembolism (VTE), especially in older patients and those with specific injuries^29^. The detrimental effects of prolonged bed rest, particularly in post-surgical patients, has long been recognized as a key risk factor for VTE^12,14^. Considering the increased risk, the National Institute of Child Health and Human Development underscored the need for proactive prophylaxis of VTE in immobilized patients^30^ and those undergoing surgery^26,27^.

#### Muscle atrophy

Disuse muscle atrophy is characterized by reduced muscle mass and functionality resulting from extended periods of muscle inactivity or immobility. Although muscle atrophy has been extensively documented^17^, the role of connective tissues, including the fascia, in the recovery process is poorly described. Fascial tissue is an essential element of the musculoskeletal system, playing a role in force transmission, structural stability, and joint function^31,32^. Without load-bearing activity, physiological studies have shown that individuals undergo a 5–20% reduction in knee extensor muscle mass after 3–4 weeks of inactivity^33–36^ and a 12–30% fall in the cross-sectional area of the knee extensor muscles after 4–16 weeks of disuse^33,37^. The extent of atrophy and metabolic adaptation can differ between muscle groups, such as the vastus lateralis and soleus, which has important implications for targeted rehabilitation strategies^3,38^. Muscle atrophy could be apparent within 2–3 days of immobilization, even in healthy subjects not undergoing surgery^3,39^. Even brief periods of immobilization of the limbs cause a pronounced decrease in muscle function and oxidative capacity, thereby complicating the rehabilitation process^21^. A systematic review and meta-analysis of 25 studies on experimental bed rest has revealed a significant decline in overall and lean body mass (effect size = −0.45, 95% c.i. –0.72 to –0.19 and −0.67, 95% c.i. –0.59 to –0.40, respectively; *P* < 0.01)^40^. In addition, the quadriceps cross-sectional area diminished significantly by 3.2% after a mere 7 days of bed rest, corresponding to a loss of 140 g thigh tissue and an 8% decrease in muscle strength (*P* < 0.01)^16^.

#### Metabolic dysregulation

Injury triggers a neuroendocrine response, marked by increased secretion of stress hormones, including adrenaline and cortisol, alongside increased release of glucagon, growth hormone, aldosterone, and antidiuretic hormone^41,42^. Surgery also impacts water, electrolyte, protein, fat, and carbohydrate metabolism^43^. Prolonged postoperative immobilization may aggravate negative nitrogen balance, insulin resistance, and muscle protein breakdown, compromising recovery^16,40^. Observations on the endocrine and metabolic responses to surgical stress underscore the need for early mobilization techniques to minimize adverse outcomes and facilitate better postoperative results^43^. Bed rest also impairs insulin-mediated suppression of fat oxidation and carbohydrate oxidation, suggesting a dissociation between glucose utilization and substrate oxidation^3^. These alterations are primarily influenced by changes in muscle mRNA and pyruvate dehydrogenase kinase 4 protein levels rather than intramyocellular lipid accumulation, indicating that the lack of muscle contraction is a significant factor in these metabolic alterations^3^.

A systematic review of 40 studies in patients with immobilised upper (18 studies) and lower (22 studies) limbs showed that deterioration in neuromuscular function was proportionate to the length of immobilization but was more pronounced after immobilization of the lower limbs than upper limbs^23^. Acute (3 days) and chronic (56 days) bed rest impair glucose disposal and substrate oxidation, with a 17% reduction in insulin-stimulated glucose disposal after 3 days and a 22% reduction after 56 days (*P* < 0.05)^3^. Chronic bed rest results in a 19% decrease in carbohydrate oxidation and a 43% suppression of insulin-mediated fat oxidation (*P* < 0.05), highlighting the detrimental effects of immobilization on metabolic function^3^. Prolonged periods of bed rest also result in morphological changes, such as a reduction in total lean body mass ranging from 2.3 to 4.4% within 1 week^16,40^. This is primarily linked to muscle disuse and the onset of systemic insulin resistance, as well as changes in hormonal and inflammatory factors^16,40^. Decreases in glucose uptake and disposal can increase susceptibility to metabolic dysfunction and compromise tissue regeneration.

Furthermore, decreased mechanical loading leads to bone demineralization, which increases the risk of osteopenia, fractures, and prolonged musculoskeletal dysfunction. Patients on bed rest experience a monthly loss of 1–2% of bone mineral density, especially in weight-bearing bones^24^.

**Fig. 2:**
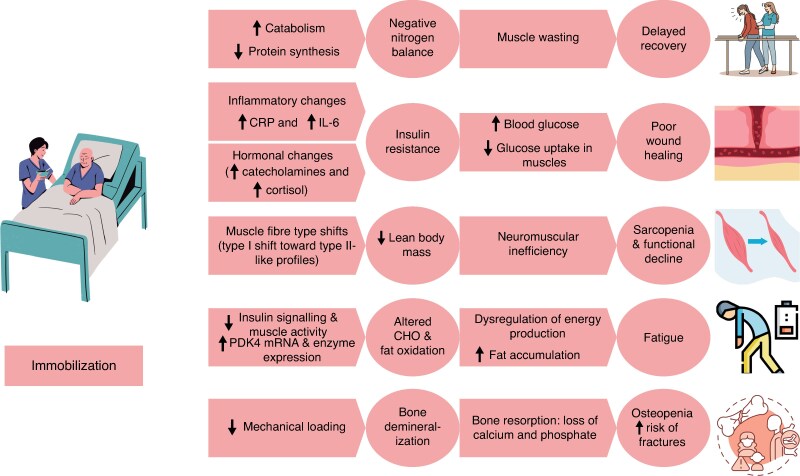
Metabolic dysregulation by and consequences of immobilization CRP, C-reactive protein; IL-6, interleukin-6; PDK4, pyruvate dehydrogenase kinase 4; CHO, carbohydrates.

The combined effects of metabolic dysregulation, muscle wasting, and decreases in bone density due to immobilization constrain postoperative rehabilitation (*Fig. 2*), highlighting the need for early mobilization strategies to avert long-term functional disability.

#### Pulmonary complications

Immobilization also results in deterioration of pulmonary function, increasing the risk of postoperative pulmonary complications (PPCs) such as atelectasis, pulmonary embolism, pneumonia, and hypoxaemia because of the decrease in lung expansion and effectiveness of secretion clearance^12,14^. A systematic review revealed that PPCs occur in approximately 12.8% of surgical patients, contributing to over two-thirds of inpatient postoperative mortality^44^. The odds of developing PPCs increase with the duration of surgery and are higher in patients undergoing emergency surgery, as well as in those undergoing abdominal (OR 4.4, 95% c.i. 2.3–8.5) and thoracic surgery (OR 11.4, 95% c.i. 4.9–26.0) compared with peripheral surgery (OR 1)^45^. Findings from an observational cohort study^46^ involving adult patients undergoing major abdominal surgery and a narrative review^47^ suggest that each additional day of postoperative bed rest correlates with an approximate threefold increase in the risk of PPCs.

A case-control study^48^ of more than 17 000 adult patients in mixed surgical settings found that those who were bedridden for more than 3 days after surgery had a 2.7-fold higher risk of pneumonia than those mobilized earlier (*P* < 0.001). Prospective observational physiological studies^49,50^ in adult postoperative surgical patients have demonstrated that simply moving from the supine to the upright position may improve blood oxygen saturation and postoperative pulmonary function.

#### Impact on LOS and functional recovery

Delayed mobilization is associated with increased LOS and poorer functional outcomes. A study^51^ with a retrospective and prospective line of research involving 125 older adults (mean age 67.2 years) undergoing surgery for degenerative scoliosis revealed that early ambulators (on the first postoperative day) experienced hospital stays that were 34% shorter, averaging 5.3 days compared with 8.1 days for those who mobilized on the second or third postoperative day. A narrative review has linked immobilization with long-term functional decline^4^, whereas a prospective cohort study^22^ of older adults undergoing major surgery demonstrated that approximately 20% experience functional impairment at 30 days after surgery. A meta-analysis involving 1941 intensive care patients indicated that prolonged immobilization significantly extended the ICU stay and LOS by 1.8 and 3.9 days, respectively^52^.

### Early postoperative mobilization and clinical outcomes

Although the physiological rationale for early postoperative mobilization is compelling, the clinical evidence supporting its effectiveness remains variable across surgical populations and outcome domains. Early mobilization is a cornerstone of enhanced recovery after surgery (ERAS) pathways, yet its stand-alone impact on clinical outcomes continues to be debated. A recent comprehensive systematic review and meta-analysis, which included 15 studies (eight RCTs) on more than 3500 patients undergoing gastrointestinal surgery, reported that early mobilization significantly accelerated gastrointestinal recovery (mean difference −11.53 h; *P* = 0.03), but did not show statistically significant improvements in morbidity rates (*P* = 0.59), postoperative mobility (*P* = 0.28), or LOS (*P* = 0.47)^53^. Similarly, a synthesis of the ERAS literature^4^ has shown that although early mobilization is commonly implemented and frequently reported as favourable, significant heterogeneity exists in study design, definitions of mobilization, and outcome measurement, thereby limiting definitive conclusions. A review^54^ of cardiac surgery cohorts found that early mobilization protocols were feasible and were associated with improved physical capacity and reduced ICU stay. However, the results varied depending on the regimen and timing of mobilization^54^. Moreover, a meta-analysis^55^ of trials in cardiac surgery showed that early postoperative mobilization improved physical function, particularly walking distance (+54.0 m) and lower limb strength, but this did not reach statistical significance. However, the effects of early postoperative mobilization on LOS and morbidity were uncertain.

Although individual trials may show benefit in specific domains or patient groups, conclusive evidence for an independent effect of early postoperative mobilization across different surgical settings remains limited. *Table 1* summarizes recent studies focusing on whole-body early postoperative mobilization, highlighting their methodologies, key outcomes, and critical limitations^56–70^.

**Table 1:** Summary of recent studies on early postoperative mobilization

Study	Types of patients	Sample size	Type of intervention	Outcomes	Critique
**Randomized clinical trials**					
Balvardi *et al*.^56^ (2021)	Colorectal surgery	99 (EM = 50; C = 49)	EM: Staff-directed assistance from a trained health professional to facilitate transfers and walking from the day of surgery, with three daily visits from POD 1, targeting at least 200 m per session, progressively increased as tolerated C: Patients educated on early postoperative mobilization, which included sitting for 2 h and staying out of bed for 6 h, with nursing assistance and encouragement for breathing exercises	Staff-directed facilitation of EM did not improve postoperative pulmonary function or reduce PPCs within an enhanced recovery pathway because there was no difference in recovery of FVC, FEV_1_, peak cough flow, or 30-day PPCs	High adherence to and activity in enhanced recovery pathways face limitations such as underpowering, exclusion of mobility-impaired patients, single-centre design, and the challenge of achieving high-distance targets Routine facilitated early postoperative mobilization may not warrant extra resources, suggesting that mobilization as tolerated is sufficient, thus challenging guidelines for intensive early postoperative mobilization
Brocki *et al*.^57^ (2016)	Pulmonary resection	70 (EM = 35; C = 35)	EM: Implemented as part of a standard postoperative physiotherapy regimen, incorporating breathing exercises, coughing techniques, and mobilization, supplemented by IMT performed twice daily (2 × 30 breaths at 30% of maximum inspiratory pressure) over a 2-week period; EM was defined as patients sitting at the bedside on the day of surgery and ambulating ≥15 m on the same day, after surgery C: Standard postoperative physiotherapy alone	EM improved oxygenation on POD 3 and POD 4 (*P* < 0.02), but there were no significant differences in respiratory muscle strength, lung volumes, functional performance, or PPCs Pneumonia incidence was 13%, with no significant difference between groups (*P* = 0.14)	A notable benefit was the trend towards reduced PPC, including atelectasis, although not statistically significant, suggesting potential clinical relevance in high-risk populations Limitations include a small sample size and possible bias in compliance reporting Clinically, IMT may be a safe adjunct to standard care, warranting larger trials to confirm efficacy in PPC prevention
Ceylan *et al*.^58^ (2024)	Cardiac surgery	100 (EM = 50; C = 50)	EM: Mobilization started on POD 1 with supervised sessions of assisted mobilization, transfers, limb exercises, and respiratory physiotherapy, conducted twice daily until discharge C: Patients could mobilize as tolerated without structured physiotherapy or scheduled sessions	EM significantly improved postoperative recovery in elderly cardiac surgery patients, reducing mechanical ventilation time (6 *versus* 10 h), ICU stay (2 *versus* 4 days), and LOS (8 *versus* 14 days) EM also led to a significant decrease in functional independence scores (−4 *versus* −11) and a greater improvement in the 6MWD (78 *versus* 37 m)	Study limitations include single-centre design, small sample size, and no long-term follow-up Uncertainty in intervention applicability across surgical populations; however, strong statistical significance and clinically relevant improvements indicate early postoperative mobilization is a beneficial, low-risk strategy for recovery and reduced healthcare use in high-risk groups
de Almeida *et al*.^59^ (2017)	Major abdominal surgery	108 (EM = 54; C = 54)	EM: Structured programme: exercises (core stability, gait, aerobic, resistance) tailored to patient mobility, starting POD 1, twice-daily sessions until discharge C: Standard rehabilitation care began on POD 1 and lasted until discharge or walking independence; daily exercises included core control, orthostatic training, gait training, and range-of-motion exercises	EM reduced the number of patients unable to ambulate independently by POD 5 or discharge (16.7 *versus* 38.9%; *P* = 0.01) The EM protocol included supervised exercises and was safe and feasible No significant differences in other outcomes or complications were noted, highlighting EM's potential for improving postoperative recovery	Clinically, the findings support integrating structured early postoperative mobilization into enhanced recovery pathways, although broader implementation requires consideration of staffing resources and patient-specific functional capacity; however, high heterogeneity in study population was observed
De Roo *et al*.^60^ (2015)	Orthopaedic surgery	130 (EM = 79; C = 51)	EM: Immediate daily passive mobilization starting directly after surgery, without any immobilization period; after surgery, patients in the EM group underwent supervised passive range-of-motion exercises daily, which included passive forward elevation, abduction in the scapular plane, external rotation with the arm at the side, and internal rotation. The protocol aimed to maintain joint mobility while protecting the rotator cuff repair site C: Gradual mobilization starting after week 4	No significant difference between the two groups regarding range of motion at 6 weeks and range of motion, strength, and functional outcome scores at 4 months Ultrasound did not show a difference in tendon healing at 6 weeks in either group	A 4-month follow-up may be too short to assess long-term tendon healing and functional recovery The lack of detail on physiotherapy intensity beyond passive mobilization is a limitation that could affect outcomes Although the findings support flexible rehabilitation protocols without compromising early results, caution is advised in generalizing these results to longer-term recovery
Dehghani *et al*.^61^ (2023)	Major laparoscopic abdominal surgery	80 (EM = 40; C = 40)	EM: Implemented as a structured programme comprising two rounds of mobilization after surgery, specifically aimed at enhancing recovery and reducing pain; the protocol involved getting surgical patients out of bed early, typically within 2–3 h after surgery on the day of the procedure or on POD 1. Pain scores were systematically assessed using the VAS before and after each mobilization session to monitor the intervention’s efficacy C: Standard postoperative care without EM sessions, including routine monitoring and activities. Pain assessments using the VAS were conducted for comparison with the intervention group	The EM group showed significant pain reduction over time (*P* < 0.05) compared with control, despite similar baseline scores (*P* = 0.95) Early mobilization is a safe, cost-effective method to reduce postoperative pain after laparoscopic surgeries	Early postoperative mobilization is cost-effective for recovery, focusing on pain intensity The study lacked broader evaluations, like LOS and complications, had a single-centre design, and a small sample size, limiting generalizability The findings support early postoperative mobilization in postoperative pain management for minimally invasive surgeries
Fiore *et al*.^62^ (2017)	Colorectal surgery	99 (EM = 50; C = 49)	EM: Defined as staff-directed facilitation involving assisted transfers and walking, initiated on the day of surgery. This protocol included three daily sessions from POD 1 to POD 3 or discharge, with each session aiming for at least one corridor lap (200 m), progressively adjusted according to patient tolerance C: Usual care (including preoperative education about early mobilization with postoperative daily targets)	The EM group achieved 66% recovery of walking capacity *versus* 57% in the control group (*P* = 0.42) No significant differences in discharge time between the EM and control groups (4.3 *versus* 4.5 days, respectively) or gastrointestinal recovery (2.8 *versus* 3.1 days, respectively), with similar 30-day CCI (16.5 *versus* 18.8, respectively; *P* = 0.53) Higher in-hospital step counts in EM group (6600 *versus* 4682) but no improved outcomes, indicating limited benefit of EM	Early postoperative mobilization increased in-hospital physical activity but did not significantly enhance recovery or discharge times compared with standard care; this suggests limited benefits from additional mobilization support, likely due to the high baseline activity in the control group Although the study questions intensive mobilization protocols, it supports a pragmatic approach of mobilization as tolerated in perioperative care
Jensen *et al*.^63^ (2015)	Radical cystectomy for bladder cancer	107 (EM = 57; C = 50)	EM: Progressive, standardized plan including scheduled time out of bed increasing from 3 h on POD 1 to 8 h by POD 4, with walking distance goals increasing from 125 m on POD 1 to 1000 m on POD 4 C: Standard fast-track care without structured rehabilitation, including walking activity and early removal of intravenous and urinary catheters	EM improved postoperative outcomes, increasing walking distance and performance of personal ADL by 1 day (*P* < 0.05), but did not affect LOS	Early postoperative mobilization enhanced physical recovery but had minimal impact on clinical outcomes like LOS or morbidity, likely due to existing fast-track pathways and surgical techniques The limited effect of early postoperative mobilization on LOS highlights the complexity of postoperative recovery
Jonsson *et al*.^64^ (2019)	Thoracic surgery	107 EM = 54 C = 53	EM: Patients provided with individualized advice on physical activity based on WHO and Swedish recommendations, emphasising the importance of achieving at least 150 min of moderate activity per week while minimizing sedentary behaviour. As part of the EM protocol, patients were instructed to perform breathing exercises consisting of 10 deep breaths with PEP three times daily until pain subsided. EM was defined as engaging in individually tailored activities commencing on the day of surgery, which included sitting up in bed or in a chair, followed by progressive mobilization on the ward from POD 1. Patients were encouraged to walk as much as possible throughout the day, with or without assistance, based on their individual capabilities and needs C: Patients received no physiotherapy during hospitalization, only standard nursing care for pain management and mobility	There were no statistically significant differences between the groups in terms of physical capacity, physical activity, spirometry values, or dyspnoea	Clinical findings indicate that routine early postoperative mobilization alone may not improve long-term functional recovery following surgery Limitations include a short therapy duration of 4–5 days, potential underpowering attributed to normal preoperative 6MWD, baseline differences between groups, and the absence of preoperative objective activity evaluation
Mihaljevic *et al*.^65^ (2024)	Major abdominal surgery	347 (EM = 174; C = 173)	EM: Patients wore a fitness tracker during their postoperative stay until discharge or for a maximum of 30 days to monitor steps, aiming for daily goals with real-time feedback. The goals included: to ambulate as permitted; exceed the previous day's steps; aim for > 4000 steps; and reach this target by POD 5 for laparoscopic or POD 8 for open surgery C: Patients wore a fitness tracker after surgery for up to 30 days without feedback and could mobilize freely, supported by care teams without specific recommendations	Wearable tracker-guided EM reduced postoperative complications (18 *versus* 30%; *P* = 0.03), increased physical activity (4500 *versus* 3200 steps; *P* = 0.01), and shortened LOS (6.2 *versus* 7.5 days; *P* = 0.04), compared with standard care	Limitations: selection bias, no blinding, reliance on step count Generalizability to other surgical settings may be limited Study provides insights on using wearable technology for patient engagement in early postoperative care within ERAS pathways
Ni *et al*.^66^ (2018)	Liver resection	119 (EM = 59; C = 60)	EM: Initiated on POD 1, with patient mobilization guided by vital signs and individual tolerance. Activity levels were progressively increased, prioritizing safety by monitoring stability and gradually enhancing walking distance and step count. To facilitate accurate tracking, patients used the Fitbit Flex to monitor steps and distance, with a focus on pain assessment and fall prevention throughout the mobilization process C: Patients received usual care on POD 2 or POD 3 without the structured EM protocol. Their activity levels were not actively encouraged or monitored with wearable devices	The EM group showed better outcomes than the control group: greater walking distance and steps (*P* < 0.05), lower pain scores, longer sleep (*P* < 0.05), quicker times to first defaecation (2.2 *versus* 3.3 days; *P* < 0.01) and first flatus (2.3 *versus* 3.1 days; *P* = 0.04), reduced LOS (6.6 *versus* 7.7 days; *P* = 0.01), and discharge criteria (5.1 *versus* 6.3 days; *P* = 0.002) No significant difference in complications	Objective monitoring enhances validity but has biases and lacks long-term assessments Integrating early postoperative mobilization in recovery protocols is beneficial
Ni *et al*.^67^ (2022)	Liver resection	42 (EM = 21; C = 21)	EM: Initiated on POD 1, following a structured activity protocol designed to enhance recovery and ensure patient safety. Activities were scheduled in three periods, with nursing staff closely supervising to mitigate potential adverse effects, such as palpitations or fatigue. The mobilization regimen commenced on the day of surgery with passive movements in bed, progressing to sitting on the bed with legs hanging down on POD 1. Mobilization outside the ward was introduced on POD 2, with gradual increases in walking frequency and duration, as tolerated. The use of the Fitbit app facilitated continuous monitoring, enabling healthcare professionals to assess physical activity levels and promptly address any symptoms. Nursing staff regularly evaluated and adjusted the mobilization plan to optimize safety and efficacy throughout the recovery process. C: Patients received routine care with postoperative activity based on individual tolerance, without a standardized protocol	The EM group showed lower pain scores, improved sleep quality, quicker gastrointestinal recovery, higher physical activity levels, and shorter LOS (*P* < 0.05), with no significant differences in complications or mortality	The study is strong due to its randomization and monitoring but has a small sample size and potential bias from family involvement in activity supervision The findings suggest early postoperative mobilization can improve recovery in hepatic surgery, but larger trials are necessary for broader applicability
Svensson-Raskh *et al*.^68^ (2021)	Abdominal surgery	138 (EM = 73; C = 65)	EM: Defined as assisting the patient in mobilizing out of bed to sit in a chair within 2 h after arrival at the postoperative recovery unit, commencing immediately following abdominal surgery and continuing for up to 6 h. This process involved a therapist and a nurse facilitating mobilization by positioning the patient in a chair or at the bedside. The therapist provided guidance to ensure correct technique while performing ten sets of three deep breathing exercises per hour, thereby promoting optimal respiratory function during the early postoperative phase C: no mobilization or breathing exercises were performed	Patients who received EM showed significant improvements in SpO_2_ and PaO_2_ (*P* < 0.05) compared with controls, with no differences in other respiratory measures or LOS	The study found no significant reduction in respiratory complications, limiting long-term benefits Study limitations include a short follow-up and exclusion of severe cases However, supporting early postoperative mobilization could improve early postoperative outcomes
**Retrospective cohort study**					
Grass *et al*.^69^ (2018)	Colorectal surgery	1170 (EM = 494; C = 676)	EM: Defined as the ability to be out of bed for at least 6 h on POD 1, in accordance with ERAS guidelines and institutional standardized care maps. Patients who successfully achieved this target implemented ERAS recommendations through a structured protocol, which promoted sitting and walking on the day of surgery and on POD 1. Mobilization time was systematically tracked via nursing records, with those not meeting the specified criterion categorized as having delayed mobilization C: Patients not meeting early mobilization targets (<6 h out of bed on POD 1) were classified as having delayed mobilization, either due to inability or lack of encouragement to mobilize as per protocol	Patients who did not mobilise early had a significantly higher major complication rate (16 *versus* 7%) and longer LOS (12 *versus* 6 days)	The findings support established ERAS guidelines; however, the retrospective observational study design has limitations, including potential confounding variables such as baseline patient frailty, age, surgical complexity, and inconsistencies in nursing documentation of mobilization time
**Non-randomized quasi-experimental study**					
Koyuncu and Iyigun^70^ (2022)	Major abdominal surgery	42 (EM = 21) C = 21	EM: Structured protocol that included preoperative evaluation and patient education, aiming to establish specific mobilization goals within 24 h after surgery. EM was defined as initiating patient mobilization on POD 1, with targeted objectives such as remaining out of bed for at least 2 h on the day of surgery, progressively increasing activity duration in accordance with ERAS protocol recommendations C: patients received standard care and mobilized later with fewer goals to achieve	The EM group had a significantly shorter time to first mobilization (373.33 *versus* 733.1 min) and quicker flatus passage (47.0 *versus* 71.6 h) EM reduced ICU stay (2 *versus* 4 days) and total LOS (7 *versus* 12 days) compared with the control group (*P* < 0.001) Sleep quality and satisfaction scores were higher in the EM than control group (median scores of 8 *versus* 4 and 9 *versus* 5, respectively; *P* < 0.001)	A structured early postoperative mobilization protocol improved patient outcomes after major abdominal surgery, leading to shorter ICU stay, faster recovery, and higher satisfaction than standard care; however, monitoring was limited to POD 1, restricting long-term insights The study findings support integrating these protocols into postoperative care

EM, early postoperative mobilization group; POD, postoperative day; m, minutes; C, control or standard care group; h, hours; PCCs, postoperative pulmonary complications; FVC, forced vital capacity; FEV_1_, forced expiratory volume in 1 second; IMT, inspiratory muscle training; ICU, intensive care unit; LOS, length of hospital stay; 6MWD, 6-minute walk test distance; VAS, Visual Analogue Scale; CCI, comprehensive complication index; ADL, activities of daily living; WHO, World Health Organization; min, minutes; PEP, positive expiratory pressure; SpO_2_, peripheral oxygen saturation; PaO_2_, arterial oxygen pressure; ERAS, enhanced recovery after surgery.

#### Postoperative complications

Despite integrating early mobilization into ERAS pathways, the impact on postoperative complications remains inconsistent, with studies reporting mixed outcomes regarding morbidity and recovery^4,53^. Among patients undergoing radical cystectomy for bladder cancer, no significant difference was observed in the severity of complications between the early mobilization and usual care groups (*P* = 0.64)^63^. Similarly, no difference was noted regarding the incidence of PPCs between the early mobilization and usual care groups (18 *versus* 24%, respectively; adjusted OR 0.67, 95% c.i. 0.23–1.99; *P* = 0.47) in patients undergoing colorectal surgery^56^.

However, some studies highlight potential benefits. After lung cancer surgery, hypoxaemia rates were significantly lower in the early postoperative mobilization group compared with the control group (15 *versus* 35%; *P* = 0.049)^57^, indicating a potentially protective effect of early mobilization. However, the overall incidence of pneumonia and clinically relevant atelectasis did not differ significantly between the two groups (*P* = 0.14 and 0.11, respectively)^57^. In addition, others have reported that postoperative complications did not differ significantly between patients who received monitoring and encouragement for early mobilization and those who did not following major abdominal surgery (*P* = 0.63)^65^.

Early postoperative mobilization promotes venous return, minimizes stasis, and reduces dependency on pharmacological prophylaxis, thereby lowering the risk of serious thromboembolic events^26,28^. In 2019, the American Society of Hematology recommended individual assessment of risk factors and implementation of prophylactic measures, such as early postoperative mobilization, to mitigate the risk of VTE in hospitalized surgical patients^26^.

#### Reduction in LOS

In a retrospective single-centre study focused on major gastrointestinal surgery^71^, mobilization starting 3 days after surgery was associated with longer hospitalization than mobilization on the first or second postoperative day (65.9 *versus* 41.9 and 47.9%, respectively). Early mobilization after orthopaedic^72^ and colorectal^69^ surgery improved functional independence and increased early discharge rates. In a recent meta-analysis that included 9076 surgical patients^73^, compliance with ERAS protocols was associated with a reduction in LOS by 1.9 days.

However, the evidence regarding the effect of early postoperative mobilization on LOS is not consistent. In one study, median LOS was 8 days in both the early postoperative mobilization and usual care groups after radical cystectomy (*P* = 0.68)^63^. In addition, there was no statistically significant difference in the mean LOS between patients who received monitoring and encouragement for early mobilization and those who did not (14 *versus* 13 days, respectively; *P* = 0.49)^65^. Moreover, another study found that the median hospital LOS did not differ significantly between groups with and without early mobilization after major abdominal cancer surgery^59^. However, a *post hoc* analysis^59^ suggested that early postoperative mobilization in some patients facilitated early discharge (LOS ≤ 7 days was recorded in 14.8% of patients in the control group and in 33.3% of patients in the early mobilization group; *P* = 0.024).

#### Quality of life

Evidence regarding the impact of early postoperative mobilization on QoL is mixed. QoL scores have been reported as comparable between patients who received monitoring and encouragement for early mobilization and those who did not at baseline and on postoperative days 6 and 8 after major abdominal surgery^65^. The similarity was consistent across all dimensions evaluated, including physical functioning, role functioning, emotional functioning, cognitive functioning, social functioning, and global health^65^. In addition, no significant differences were observed between the two groups in symptom scores related to fatigue, nausea, vomiting, pain, shortness of breath, insomnia, appetite loss, constipation, or diarrhoea^65^.

However, an RCT^67^ showed that early postoperative mobilization significantly improved sleep quality on the fifth postoperative day after liver resection compared with usual care (*P* < 0.05). Additional outcomes were decreased sleep latency, reduced sleep disturbances, lower dependence on sleep medicines, and enhanced daytime functioning in those undergoing early mobilization^67^.

#### Reduction in postoperative pain and improved functional recovery

Delays in postoperative mobilization can lead to muscle and joint stiffness and pain, whereas early postoperative mobilization has been observed to reduce musculoskeletal complications among patients undergoing laparotomy and those with unilateral lower limb suspension^7,33^. In patients undergoing hip and lower extremity orthopaedic surgery, early postoperative mobilization was associated with lower postoperative pain scores compared with prolonged immobilization^72^. An observational study in older surgical patients^22^ and a narrative review of ERAS pathways^4^ suggest that early postoperative mobilization and exercise-based activities could minimize severe postoperative deconditioning and reduce the long-term risk of functional disability.

Although early postoperative mobilization offers these general benefits, studies show mixed results regarding specific functional outcomes. No significant differences were found between the two groups regarding functional outcome scores at 4 months after surgery in an orthopaedic surgical population^60^. Similarly, among patients undergoing pulmonary resection, there were no significant differences between the early mobilization and usual care groups on postoperative day 5 or 2 weeks after surgery in terms of respiratory muscle strength, lung volumes, physical performance, dyspnoea levels, or peripheral oxygen saturation (*P* > 0.05)^57^.

However, one notable area of improvement with early mobilization was walking capacity. Among patients undergoing cardiac surgery, the early mobilization group had a significantly higher mean walking distance in 2 min than the control group (mean(s.d.) 135.6(9.3) *versus* 123.4(8.5) m, respectively; *P* < 0.001)^58^. In addition, on the fifth postoperative day after major abdominal surgery, a smaller proportion of patients in the early mobilization group required assistance to walk 3 m than those in the standard care group (16.7 *versus* 38.9%. respectively; *P* = 0.01)^59^. Moreover, that study^59^ showed that the early mobilization group had an absolute risk reduction of 22.2% (95% c.i. 5.9–38.6%) in the inability to cross the room or walk a distance of 3 m without assistance compared with standard care, with the number needed to treat of 5.

#### Patient satisfaction

In one study^60^, 4 months after orthopaedic surgery, 86% of participants in the immobilization group were satisfied, compared with 92% in the early mobilization group (*P* = 0.37). In contrast, among patients undergoing major abdominal surgery, it was observed that patients who underwent early mobilization had significantly improved ambulation capacity and reported higher satisfaction levels than those who underwent prolonged immobilization^70^. In addition, the early mobilization group had higher median sleep quality and satisfaction scores (*P* < 0.05)^70^.

#### Mortality

None of the studies reviewed reported differences in postoperative mortality between early and delayed mobilization groups.

#### Cost reduction

Early initiation of postoperative mobilization reduces LOS, resulting in lower overall healthcare costs in patients undergoing major laparoscopic abdominal surgery^61^. Economic analyses of the early mobilization approach by applying ERAS protocols have proven to have a substantial return on investment, with significant cost savings in perioperative care^74^. A multisite implementation of ERAS protocols in Alberta, Canada, demonstrated a return on investment of 7.3 : 1, equating to an average cost saving of CAD 2182 per patient^74^. In the UK, implementation of ERAS protocols for open liver resection has been associated with a cost saving of approximately £5255 per patient^75^. Minimizing the risk of developing complications such as DVT, pneumonia, and prolonged functional decline through early mobilization reduces the demand for high-level interventions and lengthy rehabilitation services. However, it is difficult to discern what proportion of the reduction in costs is directly related to early postoperative mobilization.

### Anaesthetic techniques and early postoperative mobilization

The anaesthetic management of the patient can significantly affect the success of early postoperative mobilization through effects on analgesia, cognition, motor function, and haemodynamic stability. The use of local or regional anaesthetic techniques with minimal sedation allows rapid recovery, which should facilitate early postoperative mobilization by avoiding adverse effects associated with hypnotic sedatives, opioids, and muscle relaxants and offering effective pain relief. Similarly, spinal anaesthesia has been promoted for rapid recovery^76^; however, it can potentially delay mobilization through lingering sympathetic blockade resulting in hypotension, motor weakness, and urinary retention, particularly with longer-acting local anaesthetics such as bupivacaine. Importantly, current evidence suggests no differences in outcomes between spinal anaesthesia and ‘fast-track’ general anaesthesia techniques^76,77^. An ideal ‘fast-track’ general anaesthesia technique would include avoiding the routine use of preoperative sedatives such as midazolam and avoiding deep anaesthesia^77^. Excessive sedation delays the restoration of cognition, impedes mobilization, and increases the risk of sedation-related motor dysfunction and falls^78^.

Effective pain control is also necessary for early mobilization^79^. A recent systematic review and meta-analysis^80^ that included a total of 5614 patients found that opioid-sparing strategies reduced 24-h morphine consumption, improved postoperative pain scores, and decreased side-effects associated with opioid usage, such as nausea and pruritus. Of note, routine opioid-free anaesthesia techniques have no clinical benefits, and may potentially delay mobilization through lingering sedative and hypotensive effects of analgesic adjuncts such as dexmedetomidine, ketamine, and magnesium^81^. An optimal multimodal analgesia regimen would consist of a combination of paracetamol, non-steroidal anti-inflammatory drugs, dexamethasone, and local or regional analgesic techniques, and should reduce postoperative pain and opioid requirements^82^. One of the limitations of the single-injection regional analgesia technique is the possibility of rebound pain, which can also delay mobilization. The use of paracetamol and non-steroidal anti-inflammatory drugs as scheduled (around the clock) should minimize the possibility of severe pain when the regional block wears off^82–84^.

Aggressive antiemetic prophylaxis is critical in achieving early mobilization^85^. Because adherence to risk-based prophylaxis remains inconsistent, universal antiemetic prophylaxis is recommended. All patients should receive at least two antiemetics, whereas for high-risk patients (for example, those with history of motion sickness, previous postoperative nausea and vomiting, or high opioid needs), three to four antiemetics are recommended^85^.

Postoperative delirium is another barrier to early postoperative mobilization, particularly in older adults. The rise in incidence of postoperative delirium is due to high exposure to opioids and sedatives; however, the incidence of postoperative delirium can be minimized through the implementation of multimodal analgesia that conserves opioid use^86^. Early postoperative mobilization itself appears to be protective, helping to maintain orientation, circadian rhythm, and functional capacity^87^.

### Adverse events associated with early postoperative mobilization

Early postoperative mobilization is not without attendant adverse effects, among which orthostatic intolerance, falls, and dizziness are of particular concern. Orthostatic intolerance is a common occurrence early after mobilization, especially on the day of surgery, with an incidence of as high as 22% in early mobilization groups compared with 6% in standard care groups after colorectal surgery^62^. In adult patients early postoperative orthostatic intolerance, recently described in a narrative review of perioperative care^88^, is characterized by dizziness, nausea, and syncope on standing, and may result from autonomic dysfunction, residual effects of anaesthesia, opioid therapy, and perioperative fluid redistribution. The development of early postoperative orthostatic intolerance is a considerable problem, because its treatment requires a delicate balance between promoting early mobility and maintaining patient safety^88^. Recent studies have started to investigate strategies to overcome such problems. A feasibility study^89^ in 84 patients undergoing total hip arthroplasty found that a standardized programme of repeated early postoperative mobilization could successfully overcome orthostatic intolerance within 4–6 h. Similarly, a qualitative study^90^ in colorectal surgery highlighted that although early postoperative mobilization is feasible, it is also resource intensive and often challenging for patients experiencing symptoms such as dizziness, nausea, or fatigue, underscoring the need for personalized care and adequate staffing.

Falls in the early stages of postoperative mobilization can result from imbalance, reduced lower extremity strength, and transient hypotension, and may cause injury and prolong the duration of recovery^62,69,91^. Episodes of dizziness are common with positional changes and can be exacerbated by inadequate fluid replenishment or the effects of analgesia and anaesthesia during and after major surgery^69,91,92^. Although these risks are interrelated, the overall incidence of falls and serious adverse effects is relatively low when mobilization occurs with correct supervision and effective risk-prevention measures^93^.

### Limitations of studies on early postoperative mobilization and future challenges

**Fig. 3:**
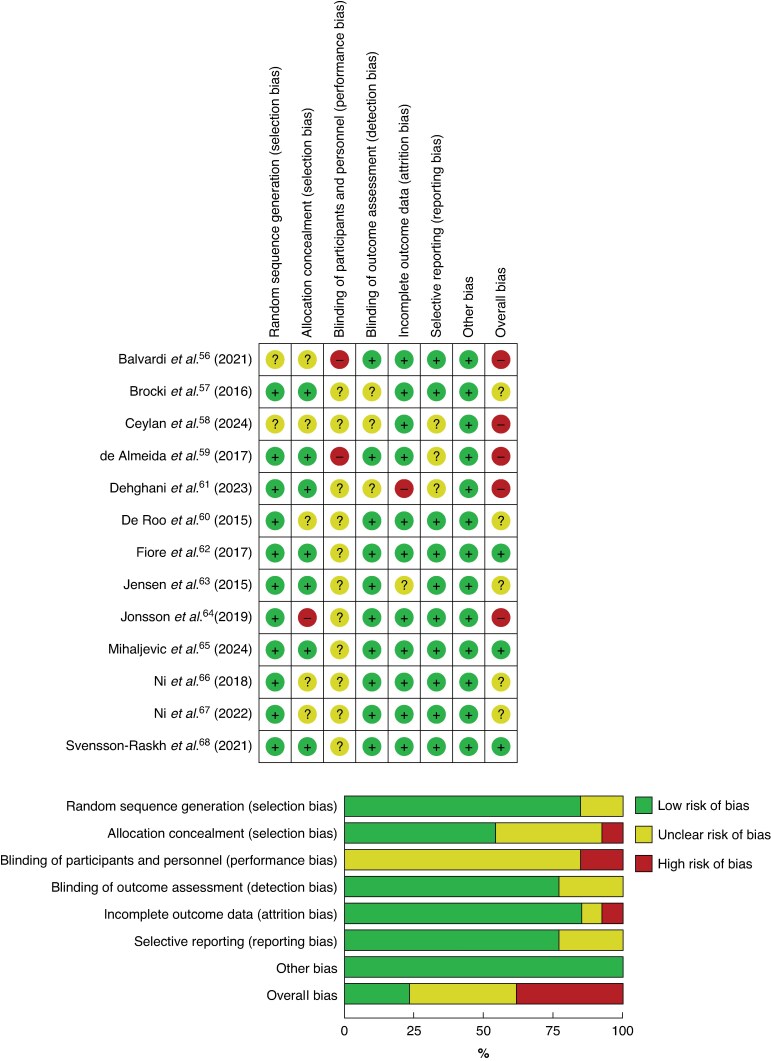
Quality assessment of randomized clinical trials

Despite the increasing evidence supporting early postoperative mobilization, several factors limit the generalizability and interpretation of study outcomes. First, a serious limitation concerns the methodological quality of the studies included. *Figure 3* summarizes the quality assessment, indicating that although most RCTs showed low risk in randomization, reporting, and outcome assessment, concerns were raised in several studies related to allocation concealment, the absence of blinding, adherence to protocols, and biases in reporting specific outcome results. Nevertheless, blinding and allocation concealment are difficult to achieve in such studies. Several studies were conducted at single centres with small numbers of participants and had limited follow-up periods. Overall, most of the studies have been classified as having a high risk of bias or having some concerns. Beyond methodological limitations, a fundamental issue is the lack of a standard definition of early postoperative mobilization, because protocols vary widely between studies. For example, some trials define mobilization as assisted transfers and walking within the first postoperative day^62^, whereas others include structured exercise programmes or prolonged out-of-bed time^58,59^, leading to inconsistency and difficulties making comparisons across studies. Furthermore, population heterogeneity poses a significant challenge, with studies encompassing diverse surgical specialities (for example, orthopaedic, abdominal, cardiac) and patient characteristics (for example, age, co-morbidity profiles), resulting in variable responses to mobilization protocols^63,65^. In addition, early postoperative mobilization regimens differ in intensity, frequency, and duration, ranging from brief assisted walks^57^ to comprehensive physiotherapy programmes^63^, making determining the optimal mobilization strategy difficult. For example, hospitals also vary in their recommendations: King’s College Hospital NHS Trust advises step targets of 250 steps/day on postoperative day 1 and 1250 steps/day by day 4 following colorectal surgery^94^, whereas Oxford University Hospitals NHS Foundation Trust recommends walking 60 m (∼80 steps) on day 1 and 200 m (∼260 steps) on day 3 after debulking surgery for ovarian cancer^95^. Another example is the recent ERAS^®^ Society recommendation that patients should spend more than 3 h out of bed during hospitalization after colorectal surgery^96^, but without specific documentation of reasons for this time frame. Taken together, this heterogeneity in settings, populations, and mobilization protocols results in variable certainty of evidence across surgical subspecialities, with higher-quality evidence in some and lower-quality evidence in others. As a consequence, a formal subspeciality-specific GRADE synthesis would be recommended, but this was beyond the scope of the present narrative review. To enable more robust comparative evaluation, future research should focus on standardized definitions of early postoperative mobilization and subspeciality-specific outcome measures.

Another notable limitation is the variable documentation regarding the management of patients who were either unable or unwilling to mobilize. A limited number of studies outlined criteria for withholding mobilization. For example, mobilization was stopped in the event of dizziness, palpitations, desaturation, or tachycardia^66^. Conversely, mobilization commenced only when pain and vital signs met established safety thresholds^61^. In addition, some studies excluded patients who were unable to tolerate or complete the prescribed mobilization doses^58,70^. Most of the studies did not report data on refusal or intolerance procedures, which may introduce self-selection bias, because patients experiencing early complications were more likely to stay within the low-mobility groups. This restricts the interpretability of comparative outcomes.

Outcome measures also vary, with studies focusing on diverse endpoints such as LOS, functional capacity, respiratory parameters, or patient satisfaction, complicating meta-analytical synthesis and evidence-based recommendations^56,64^. Another significant limitation of the current literature is the primary emphasis on in-hospital mobilization, with insufficient consideration of the post-discharge phase. Data obtained from accelerometer-based studies indicate that postoperative physical activity frequently remains significantly lower than preoperative levels for the early discharge period, 1 week^97^, and several weeks^98,99^ to months^100^ after surgery, which may diminish the advantages of early mobilization^101^. Post-discharge step counts often decrease and do not reliably revert to baseline levels, even after several weeks^98,100,101^.

Wearable technologies are becoming increasingly important tools in enabling patients’ recovery after discharge from hospital, especially when questioning ‘Why not fully active?’^90,97^. Interventions involving the use of these devices can increase long-term levels of physical activity and enhance patient-reported outcomes after lung cancer surgery, thus supporting the maintenance of mobility in the postoperative period^102^. A recent systematic review of RCTs on total knee arthroplasty revealed that the use of wearable (for example, neuromuscular electrical stimulation, activity sensors) and mobile applications supports postoperative mobilization, with benefits such as enhanced functional outcomes, better gait performance, increased patient satisfaction, and earlier postoperative mobilization, in addition to proper pain control and a potential reduction in costs^103^. This suggests that the absence of structured post-discharge support and pain management strategies may lead to the loss of gains achieved during the early recovery phase, underscoring the need for continued mobilization strategies in the post-discharge phase. Addressing these limitations requires establishing standardized definitions, tailored regimens, and consistent outcome and follow-up measures to enhance the robustness and clinical relevance of future studies.

### Barriers to and enablers of early mobilization

Many barriers hinder the successful implementation of early postoperative mobilization. Patient-related factors such as haemodynamic instability, excessive sedation, and postoperative pain effectively delay mobilization^28,78^. The literature suggests that haemodynamic instability (86%) and excessive sedation (63%) are among the leading causes of delays in mobilization in critically ill patients^78^. These barriers illustrate a complex interference between physiological constraints and clinical approaches. These approaches require a sensitive balance between safety measures and the degree of mobility achieved in the early phases, compared with the predicted adverse outcomes resulting from delayed mobilization.

Early postoperative orthostatic intolerance has been a significant yet commonly unnoticed barrier^88^. The management of orthostatic intolerance requires clinical acumen, and objective evaluation aids in the early identification of such individuals, permitting adjustment of mobilization plans.

Healthcare provider-related barriers, such as lack of awareness, limited educational opportunities, and resistance to prioritizing mobilization, further compound the disparities in its implementation^4^. Organizational factors such as low staffing ratios, heavy workloads, and the lack of standardized protocols also constitute system-based barriers to early postoperative movement^74^, as well as conservative activity attitudes in patients and in care advice^90^.

**Fig. 4:**
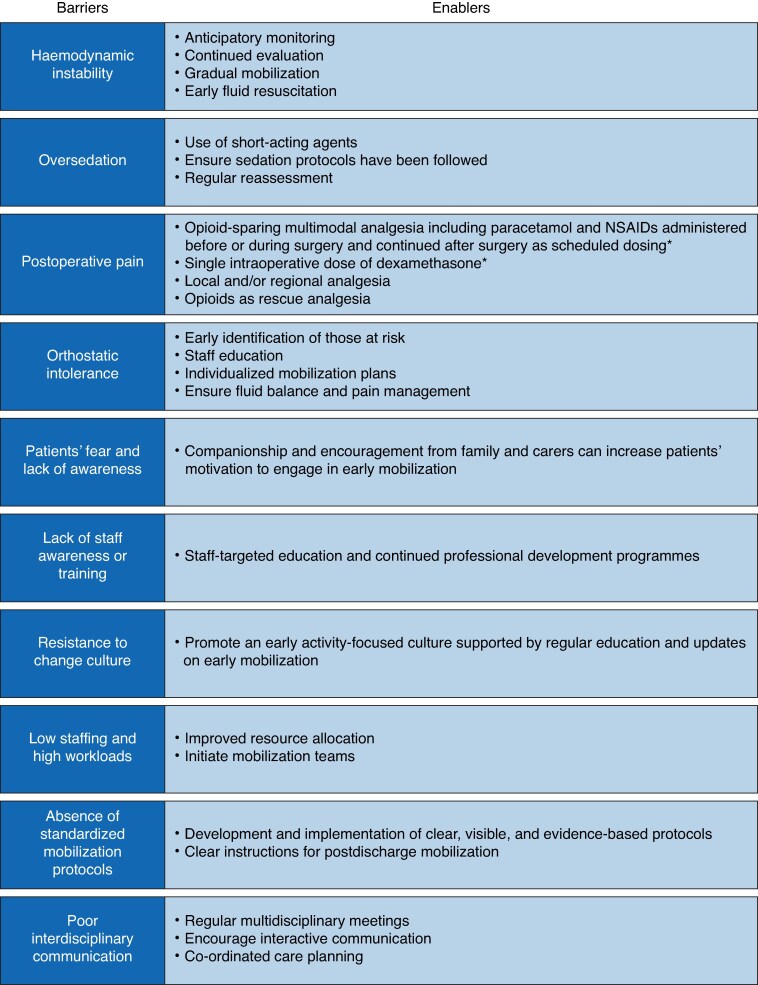
Barriers to and enablers of early mobilization *Unless contraindicated. NSAIDs, non-steroidal anti-inflammatory drugs.

Nevertheless, several facilitators can promote mobilization practices. These include improved interdisciplinary communication between surgeons, physiotherapists, and nursing staff, which has resulted in greater patient compliance with early mobilization protocols^51^, adequate pain control with opioid-sparing strategies^104^, and the promotion of an early activity-valued unit culture. Anticipatory identification and management of physiological barriers such as orthostatic intolerance are essential for safe and extended mobilization. Where early mobilization is not feasible, adjunctive measures may be considered due to factors such as pain, fatigue, or catheterization. Neuromuscular electrical stimulation has been proposed as a temporary alternative to maintain muscle function and enhance circulation until active mobilization is achievable^105^. *Figure 4* offers a structured overview of the barriers to early postoperative mobilization with suggested facilitators.

## Conclusion

The physiological consequences of prolonged immobilization, including muscle atrophy, metabolic dysregulation, and increased risk of VTE, and pulmonary morbidity, underscore the need for timely postoperative mobilization. Implementing early postoperative mobilization as a core component of ERAS protocols is essential for optimizing postoperative patient outcomes. Although early postoperative mobilization is associated with reduced LOS and improved functional recovery, the recent evidence regarding its impact on postoperative complications and overall clinical outcomes is inconsistent across various surgical populations. The dangers of bed rest and immobilization have been aptly summarized by Asher^106^ when he wrote:

*Teach us to live that we may dread*
*Unnecessary time in bed.*
*Get people up, and we may save*
*Our patients from an early grave*.

In conclusion, early postoperative mobilization offers substantial potential benefits. Importantly, the certainty of evidence supporting early postoperative mobilization differs across surgical subspecialties, emphasizing the need for future subspeciality-specific, higher-quality studies using standardized definitions of early postoperative mobilization and outcome measures. Addressing the identified barriers and leveraging facilitators will be essential to optimizing the implementation of early postoperative mobilization strategies, ultimately improving surgical recovery and patient satisfaction.

## Supplementary Material

zrag016_Supplementary_Data

## Data Availability

No original data to share.
