# Supplementary material for: Importance of early postoperative mobilization: comprehensive review
Source: BJS Open. 2026 Mar 24;10(2):zrag016. doi: 10.1093/bjsopen/zrag016 (PMC13010080; doi:10.1093/bjsopen/zrag016)
Supplement: zrag016_Supplementary_Data [file zrag016_supplementary_data.docx]

**The importance of early postoperative mobilisation: a comprehensive review**

Abdulaziz Alsuwaylihi^1,2,3^, Dominic O’Connor^4^, Girish P Joshi^5^, Henrik Kehlet^6^, Dileep N Lobo^1,2,7,8^

^1^Nottingham Digestive Diseases Centre, Division of Translational Medical Sciences, School of Medicine, University of Nottingham, Queen’s Medical Centre, Nottingham, UK

^2^National Institute for Health Research Nottingham Biomedical Research Centre, Nottingham University Hospitals and University of Nottingham, Queen’s Medical Centre, Nottingham, UK

^3^Department of Clinical Nutrition, King Saud Medical City, Ministry of Health, Riyadh, Saudi Arabia

^4^School of Health Sciences, University of Nottingham, Queen’s Medical Centre, Nottingham, UK

^5^Department of Anesthesiology and Pain Management, University of Texas Southwestern Medical Center, Dallas, Texas, USA

^6^Section for Surgical Pathophysiology, Rigshospitalet, Copenhagen University, Copenhagen, Denmark

^7^MRC Versus Arthritis Centre for Musculoskeletal Ageing Research, School of Life Sciences, University of Nottingham, Queen’s Medical Centre, Nottingham, UK

^8^Divison of Surgery, Perelman School of Medicine, University of Pennsylvania, Philadelphia, PA, USA

**Corresponding author.**

Professor D. N. Lobo

Nottingham Digestive Diseases Centre

National Institute for Health Research (NIHR) Nottingham Biomedical Research Centre

Nottingham University Hospitals NHS Trust and University of Nottingham

Queen’s Medical Centre, Nottingham NG7 2UH, UK

Tel: +44-115-8231155

Fax: +44-115-8231160

Email: [Dileep.Lobo@nottingham.ac.uk](mailto:Dileep.Lobo@nottingham.ac.uk)

**ORCID ID:** 0000-0003-1187-5796;

**Twitter**: @DL08OMD

**Supplementary Materials - Index**

| **Supplementary Figures and Tables** |  |
| --- | --- |
| Supplementary Table 1 | *page 2-4* |
| **References** | *page 5* |
|  |  |

**Supplementary Table 1: Historical publications on early postoperative mobilisation**

| **Authors** | **Main findings related to early mobilisation/ immobilisation** | **Additional Comments** |
| --- | --- | --- |
| McDowell 1819^1^ | - Benefits of early postoperative mobilisation of the patient, evidenced by the patient engaging in activities such as making her bed and travelling 70 miles on horseback just 25 days after surgery. | - The earliest evidence of potential benefits of early mobilisation |
| Ries 1899^2^ | - Early mobilisation following abdominal surgeries, such as celiotomy and vaginal celiotomy, was critical in preventing muscular atrophy, enhancing strength recovery, and facilitating earlier discharge, typically within 12 days of surgery. - The author advocated against strict immobilisation, emphasising that even minimal movement can significantly improve patient outcomes and expedite the return to normal activities. | - Early mobilisation reduced wound complications if infection prevention measures were taken. - The beneficial effects of this strategy have led some practitioners to adopt comparable strategies with satisfaction. |
| Boldt 1907^3^ | - The findings emphasised that early mobilisation was crucial in mitigating postoperative complications such as venous stasis and lung congestion. At the same time, immobilisation was linked to adverse outcomes, including impaired circulation and increased infection risk. - The report advocated for gradual, patient-specific activity protocols to enhance recovery while ensuring safety. | – |
| Leithauser and Bergo 1941^4^ | - Early mobilisation significantly enhanced postoperative recovery, especially on the first postoperative day. - Early mobilisation reduced complications like pneumonia and thrombophlebitis, the duration of hospitalisation and the period of disability. It also enhanced circulation and accelerated recovery. - Early mobilisation was contraindicated in severe abdominal distension, rigidity, or unsecured wounds, requiring a cautious and supervised approach. - Early mobilisation was safe and cost-effective. | - The first published evidence of the advantages of early mobilisation on postoperative day 1. - The success of early mobilisation relied on patient selection based on mental attitude and well-being, suggesting that not all patients were appropriate for this approach. |
| Leithauser 1943^5^ | - Early postoperative mobilisation was essential for facilitating rapid recovery and restoring functional capacity, particularly in military contexts where swift rehabilitation is critical. - Early mobilisation, ideally initiated within the first 24 h after anaesthesia, significantly enhanced vital capacity and accelerated recovery. It overcame traditional barriers rooted in fear rather than physiological limitations from surgeons and patients. | - Over 98% of patients responded favourably to early mobilisation, indicating high patient acceptance and safety of the practice. - Despite prior research, early mobilisation still faced pushback. |
| Dock 1944^6^ | - Early mobilisation fostered improved patient morale by alleviating fear and apprehension associated with surgery, facilitated earlier discharge, and optimised hospital resource utilisation while decreasing healthcare costs. | - Advantages of early mobilisation on psychosocial outcomes. |
| Ghormley 1944^7^ | - Early internal fixation for intertrochanteric fractures and other skeletal injuries significantly reduced bed rest and hospitalisation, thereby enhancing patient outcomes and well-being. - Strategies that promoted early mobilisation, such as pin fixation for fractured femurs, underscored the importance of minimising enforced rest to mitigate the risks associated with prolonged immobilisation. | - Advantages of early mobilisation in patients with fractures. |
| Harrison 1944^8^ | - Strict immobilisation after myocardial injury might increase mortality and was not recommended for patients with congestive heart failure or myocardial infarction. - Prolonged bed rest could exacerbate psychological distress and elevate the risk of complications such as thrombus formation and subsequent cardiovascular events. | - At that time, physical activity would still have been considered risky for patients with cardiac diseases. |
| Nelson 1944^9^ | - The efficacy of early mobilisation was mainly dependent upon appropriate patient selection. - Early mobilisation was not recommended for patients with wound complications, substantial debility, or thoracolumbar sympathectomy in the second stage. | – |
| Powers 1944^10^ | - Early postoperative mobilisation reduced duration of convalescence and complications, including atelectasis and pneumonia, improving rehabilitation outcomes. - It mitigated gastrointestinal dysfunction, prevented muscle atrophy and bone demineralisation, and facilitated a quicker return to everyday dietary habits and activities. | - The first study to report data on safety and benefits of early mobilisation. |
| Ashkins 1945^11^ | - Early mobilisation enhanced patient independence and minimised anxiety, preserving physiological functions and counteracting the adverse consequences of bed rest, as shown in previous studies. | – |
| Rusk and Taylor 1945^12^ | - Early mobilisation and retraining could prevent muscular atrophy in immobilised orthopaedic patients. This speeded up functional recovery, often in half the typical recovery time. - Purposeful and progressive exercises should commence promptly, starting with bed exercises and advancing to increased physical activity. | - Priority on military readiness could compromise patient autonomy. - Psychological readiness received less focus in the early rehabilitation stages. |
| Canavarro 1946^13^ | - Early postoperative mobilisation was associated with enhanced recovery outcomes, including reduced complications such as deep vein thrombosis and pulmonary complications, shorter hospital stays, and a quicker return to normal activities. | - The author reported that some patients might be reluctant or unable to participate due to pain or fear of injury. - At that time, there were concerns that early mobilisation may cause patients pain or discomfort and hinder recovery if not properly managed. |
| Deitrick, *et al*. 1948^14^ | - Immobilisation led to significant metabolic and physiological alterations, including decreased circulatory capacity, muscle strength decline, and reduced blood volume. It impaired exercise tolerance and increased resting heart rate. - Immobilisation was associated with increased nitrogen and variable calcium excretion, indicating profound metabolic changes similar to those seen after fractures. | - The first study that reported data focused on metabolic alterations linked to immobilisation. |
| Knudson and Davis 1949^15^ | - Early mobilisation was essential in preventing deconditioning and preserving vital reflexes for daily activities. - Early mobilisation facilitated corrective therapy that promoted purposeful engagement and enhanced overall physical function. - Early mobilisation objectives encompassed alleviating discomfort, restoring confidence, and improving coordination, muscle tone, and strength. | – |
| Whedon, *et al*. 1949 ^16^ | - Immobilisation in an oscillating bed minimised muscle strength decline and catabolic changes, promoting earlier psychological readiness for mobilisation and recovery compared with fixed bed immobilisation. - Immobilisation in an oscillating bed showed a metabolic effect by preserving creatine metabolism and circulation mechanisms, resulting in reduced phosphorus loss and smaller muscle mass and strength reductions. | - The first use of technology during that period involved the oscillating bed. - It suggested complex and poorly understood metabolic responses to immobilisation and the intervention. |
| Cantor 1951^17^ | - Early postoperative mobilisation significantly reduced complications related to prolonged bed rest, such as pneumonia and venous thromboembolism, while mitigating physiological and psychological disturbances associated with hospitalisation. - Early mobilisation enhanced resource utilisation and reduced hospital costs, facilitated by advancements like caudal analgesia, which enabled effective muscle relaxation for extensive procedures. | – |
| Moss and Dohan 1958^18^ | - There was significant variation among physicians regarding the appropriate duration of postoperative convalescence. - Surgeons recommend earlier return to work compared with industrial physicians and general practitioners. - Military studies showed that shorter convalescence periods were feasible without adverse effects on recovery. - Evidence suggested that recovery times could be safely reduced for uncomplicated surgical procedures. | - The authors noted a significant disparity in opinions among various groups of physicians regarding the appropriate duration of postoperative convalescence, resulting in varied practices and recommendations. |

**References**

1. McDowell E. Observations on diseased ovaria. *Eclectic Repertory and Analytical Review: Medical and Philosophical* 1819;**9**: 546-553.

2. Ries E. Some radical changes in the after-treatment of celiotomy cases. *JAMA* 1899;**33**(8): 454-456.

3. Boldt HJ. The management of laparotomy patients and their modified after treatment. *N Y Med J* 1907;**85**: 145-153.

4. Leithauser DJ, Bergo HL. Early rising and ambulatory activity after operation - A means of preventing complications. *Arch Surg-Chicago* 1941;**42**(6): 1086-1093.

5. Leithauser DJ. Confinement to bed for only twenty-four hours after operation - A means of preventing pulmonary and circulatory complications and of shortening the period of convalescence. *Arch Surg-Chicago* 1943;**47**(2): 203-215.

6. Dock W. The evil sequelae of complete bed rest. *JAMA* 1944;**125**(16): 1083-1085.

7. Ghormley RK. The abuse of rest in bed in orthopedic surgery. *JAMA* 1944;**125**(16): 1085-1087.

8. Harrison TR. Abuse of rest as a therapeutic measure for patients with cardiovascular disease. *JAMA* 1944;**125**(16): 1075-1077.

9. Nelson H. Early ambulation following section of the anterior abdominal wall - An analysis of four hundred and twenty-six personally conducted cases. *Arch Surg-Chicago* 1944;**49**(1): 1-8.

10. Powers JH. The abuse of rest as a therapeutic measure in surgery - early postoperative activity and rehabilitation. *JAMA* 1944;**125**(16): 1079-1083.

11. Ashkins J. Early rising after surgical operations. *N Engl J Med* 1945;**233**(2): 33-37.

12. Rusk HA, Taylor EJ. Army Air Forces convalescent training program. *Ann Am Acad Pol Soc Sci* 1945;**239**: 53-59.

13. Canavarro K. Early postoperative ambulation. *Ann Surg* 1946;**124**(2): 180-181.

14. Deitrick JE, Whedon GD, Shorr E. Effects of immobilization upon various metabolic and physiologic functions of normal men. *Am J Med* 1948;**4**(1): 3-36.

15. Knudson AB, Davis JE. Medically prescribed exercises for neuropsychiatric patients; the Veterans Administration program. *JAMA* 1949;**140**(13): 1090-1095.

16. Whedon GD, Deitrick JE, Shorr E, Toscani V, Davis VB, Stevens E. Modification of the effects of immobilization upon metabolic and physiologic functions of normal men by the use of an oscillating bed. *Am J Med* 1949;**6**(6): 684-711.

17. Cantor AJ. Immediate ambulation. *Am J Dig Dis* 1951;**18**(2): 56-59.

18. Moss NH, Dohan FC. Surgical convalescence: when does it end. *Ann N Y Acad Sci* 1958;**73**(2): 455-464.
